# Global status and research trends of cuprotosis research: A bibliometrics study via CiteSpace

**DOI:** 10.1097/MD.0000000000034020

**Published:** 2023-06-16

**Authors:** Xiaoli Xie, Li Liu

**Affiliations:** a Taishan Nursing Vocational College, Taian, Shandong Province, China; b Shandong Medical College, Jinan, Shandong Province, China.

**Keywords:** bibliometric analysis, CiteSpace, copper-induced cell death, cuprotosis

## Abstract

Cuproptosis, a novel copper ion-dependent cell death type being regulated in cells, has raised concerns but lacks scientific analysis. Therefore, this study aimed to analyze the global status and emerging trends in cuprotosis research using bibliometric methods. Publications related to cuprotosis were systematically retrieved from the Web of Science Core Collection and then screened according to the inclusion criteria. Next, CiteSpace and Microsoft Excel 2021 were used to measure and visualize annual publications, categories, journals, countries, institutions, authors, co-cited references, and keywords to identify future global status and trends. A total of 2776 publications on cuprotosis were included, and the overall trend in the number of publications exhibited a rapid increase over the years. Biochemistry and Molecular Biology is the most common category, whereas the Journal of Inorganic Biochemistry is the most active. The United States is the country that produces the most articles, and University of Melbourne in Australia is the core institution involved in this field. Furthermore, Chan Pak of Stanford University is the most prolific author. Oxidative stress and antioxidant, the toxicity of copper in vitro, anticancer mechanism, and brain injury in neurological diseases are hot topics. The research frontiers are copper complexes, anticancer activity, DeoxyriboNucleic Acid binding, inflammation, and nanoparticles. This study provides the current status and trends in cuprotosis research. It may help researchers to identify hot topics and get ideas for future research directions in this field, focusing on copper complexes, anticancer activity, DeoxyriboNucleic Acid binding, inflammation, and nanoparticles.

## 1. Introduction

Cuproptosis is a novel copper ion-dependent cell death mode being regulated in cells, and it is quite different from the known cell death mechanisms such as apoptosis, pyroptosis, necroptosis, and ferroptosis.^[1–3]^ Although the concept of cuprotosis was only recently put forward in 2022, relevant research has been carried out for many years.^[4]^ It is worth noting that copper is a trace element in the human body, and thus the concentration of copper ions in cells is maintained at a shallow level through the active steady-state mechanism. Once the threshold is exceeded, copper will become toxic and lead to cell death. Several studies have reported that cuproptosis plays a role in signal transduction and modulates the etiology, severity, and progression of cancer diseases.^[5–7]^ Serving as an intervention target for copper metabolism dysfunction, cuprotosis may also provide a new antitumor method. However, to date, the mechanism of cuproptosis is still unclear.

CiteSpace software is a scientific and standardized document visualization analysis software that can objectively display the development trend and hot spots of the literature in a certain time period of time.^[8]^ In this study, we systematically collected the relevant literature on cuproptosis, applied the CiteSpace software combined with bibliometric methods to analyze the research results, and then discussed the research trends and hot spots, with the overarching goal of providing a reference for further research in this field.

## 2. Material and Methods

### 2.1. Source database and data collection

**Figure 1.**
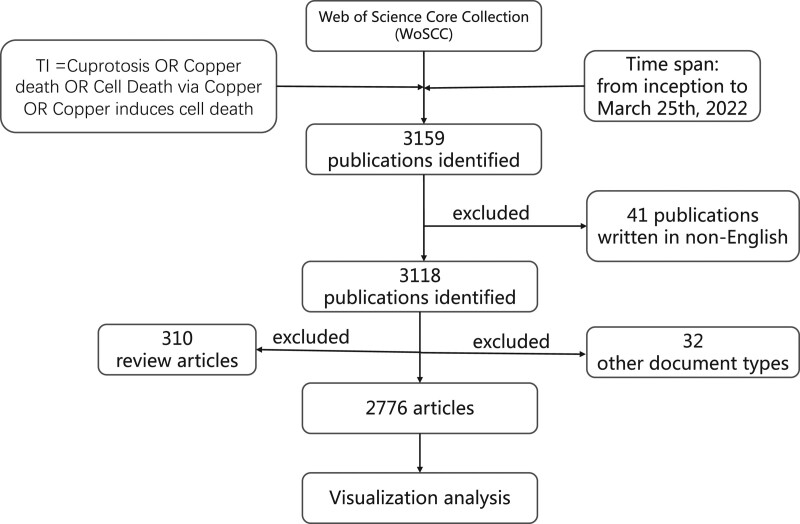
A study flow diagram of Cuprotosis from WoSCC. WoSCC = web of science core collection.

Given that the Web of Science (WoS) Core Collection has been commonly used as the source database in bibliometric analysis, we chose it for data retrieval. We systematically retrieved all relevant studies published from the inception of the WoS database up to 25^th^ March 2022. The following search strategy was applied: TI = “Cuprotosis” OR “Copper death” OR “Cell Death via Copper” OR “Copper induces cell death.” The language was restricted to English, and the document type was limited to Article. All other document types were excluded. Search results were downloaded with the record content of “Full Record and Cited References” and “Plain Text” file format. Finally, the files were renamed and imported into CiteSpace for further analysis. Figure 1 shows the flow diagram of the study. The ethical approval was unnecessary because the data do not contain any privacy information about patients.

### 2.2. Data analysis and visualization

All statistical analyses were performed using Microsoft Office Excel 2021 and CiteSpace v5.8.R2. Specifically, Microsoft Office Excel 2021 was applied to analyze the annual publication output and trend, whereas CiteSpace was used to produce visualized maps to explore the categories, journals, countries, institutions, authors, co-cited references, keywords, and burst detection for global status, and emerging trends in cuprotosis research.

## 3. Results

### 3.1. Analysis of annual publication

**Figure 2.**
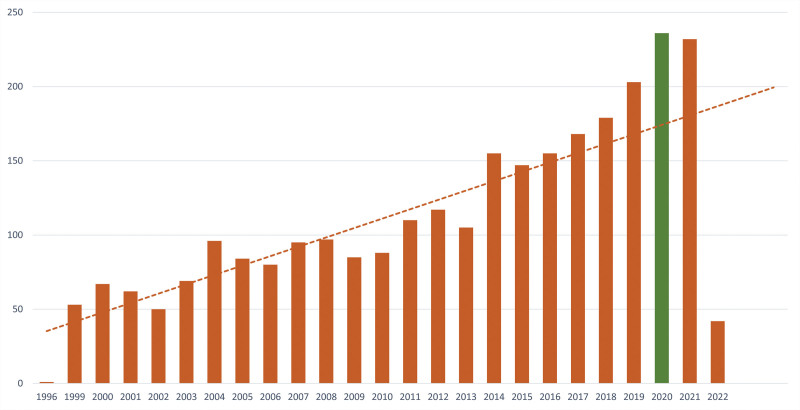
Annual publications on cuprotosis research. The horizontal coordinates represent the year of publication, whereas the vertical coordinates represent the number of publications.

Annual publications were analyzed to reflect the degree of concern and importance of cuprotosis research. This study included a total of 2776 publications (articles only). Results showed that the distribution of annual publications varied at different times (Fig. 2). It was found that the number of publications per year exhibited an upward trend from 1996 to 2022. Still, there were fluctuations in the number of publications issued in individual years (for example, 2001–2002, 2004–2006, 2008–2009, 2012–2013, 2014–2015, and 2020–2021). Overall, the evolution of published papers on cuprotosis can be divided into 3 stages: the first stage was from 1996 to 2014, which exhibited an unsteady rate of increase; the second stage was from 2015 to 2020, in which publication outputs increased gradually; and the final step was from 2021 to date, where there seems to be a downward trend of publication outputs.

### 3.2. Analysis of research categories

**Figure 3.**
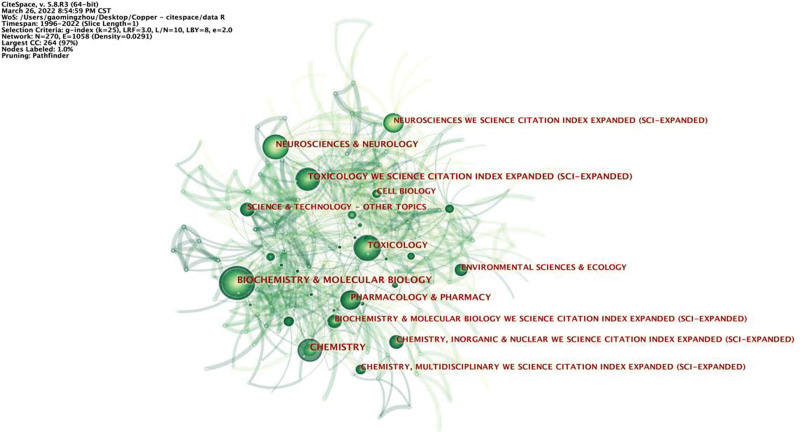
Publication distribution among different categories in the field of cuprotosis research.

Research categories were analyzed to reflect the category of cuprotosis research and help readers find relevant research more efficiently. CiteSpace was utilized to generate a category map which resulted in 270 nodes, indicating that a total of 270 categories were involved in this research field (Fig. 3). It was evident that the most frequently occurring category was Biochemistry & Molecular Biology, which was the largest circle with a frequency of 641 publications, followed by Chemistry (542 publications), Neurosciences & Neurology (283 publications), Toxicology (264 publications), and Pharmacology & Pharmacy (250 publications). Among them, Conference Proceedings Citation Index - Science presented the highest centrality value of 0.48 (Table 1).

**Table 1:** Top 10 categories associated with cuprotosis research with regard to counting and centrality.

Rank	Category	Year	Count	Category	Year	Centrality
1	Biochemistry & molecular biology	1999	641	Conference proceedings citation index - science (CPCI-S)	1999	0.48
2	Chemistry	2000	542	Science citation index expanded (sci-expanded)	1999	0.2
3	Neurosciences & neurology	1999	283	Biochemistry & molecular biology	1999	0.16
4	Toxicology	1999	264	Cell biology	1999	0.15
5	Pharmacology & pharmacy	1996	250	Biotechnology & applied microbiology	1999	0.13
6	Toxicology we science citation index expanded (sci-expanded)	1999	245	Social science citation index (SSCI)	1999	0.12
7	Neurosciences we science citation index expanded (sci-expanded)	1999	226	Engineering	1999	0.11
8	Chemistry, inorganic & nuclear we science citation index expanded (sci-expanded)	2000	203	Chemistry, applied	2000	0.08
9	Science & technology - other topics	1999	197	Toxicology	1999	0.08
10	Environmental sciences & ecology	1999	192	Chemistry, analytical	2004	0.08

**Figure 4.**
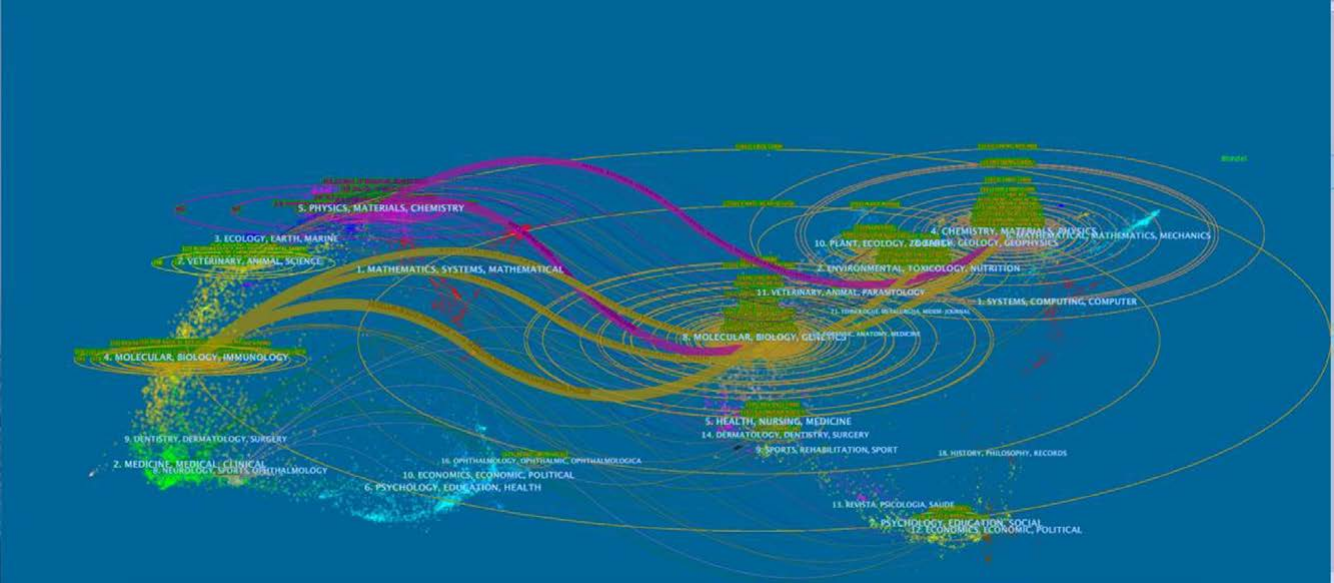
Dual-map overlay of journals publishing cuprotosis research.

Furthermore, a dual-map overlay of the journals was designed to visualize citation links from a global citing base map to a global cited base map, as illustrated in Figure 4. Referential links originated from a journal on the left of the map and pointed to a journal on the right. Notably, the color of a link distinguishes the discipline of the source. This study identified 5 main citation paths colored orange and claret, indicating that the studies published in chemistry, materials, and physics journals were mainly cited by the studies published in physics, materials, and chemistry journals. In addition, chemistry, materials, physics, environmental, toxicology, nutrition, molecular, biology, and generics journals were mainly cited by the studies published in molecular, biology, and immunology journals (Fig. 4).

### 3.3. Analysis of active journals

Analysis of journals was performed to reflect the “Core journals” with high publication and co-citation counts. Table 2 shows the top 10 journals where cuprotosis research is published. Among them, the *Journal of Inorganic Biochemistry* was the most productive journal (60 publications), followed by PLOS ONE (53 publications), *Dalton Transactions* (44 publications), *Aquatic Toxicology* (34 publications), *Free Radical Biology and Medicine* (34 publications), *Journal of Biological Chemistry* (34 publications), *Biochemical and Biophysical Research Communications* (31 publications), *Biological Trace Element Research* (31 publications), *RSC Advances* (29 publications), and *Journal of Neurochemistry* (28 publications).

**Table 2:** The top 10 academic journals publishing cuprotosis research.

Rank	Publications	Journal	IF	JCR
1	60	*Journal of Inorganic Biochemistry*	4.155	Q1
2	53	*Plos One*	3.24	Q2
3	44	*Dalton Transactions*	4.39	Q1
4	34	*Aquatic Toxicology*	4.964	Q1
5	34	*Free Radical Biology and Medicine*	7.376	Q1
6	34	*Journal of Biological Chemistry*	5.157	Q2
7	31	*Biochemical and Biophysical Research Communications*	3.575	Q3
8	31	*Biological Trace Element Research*	3.738	Q2
9	29	*Rsc Advances*	3.361	Q2
10	28	*Journal of Neurochemistry*	5.372	Q2

IF = impact factor.

**Figure 5.**
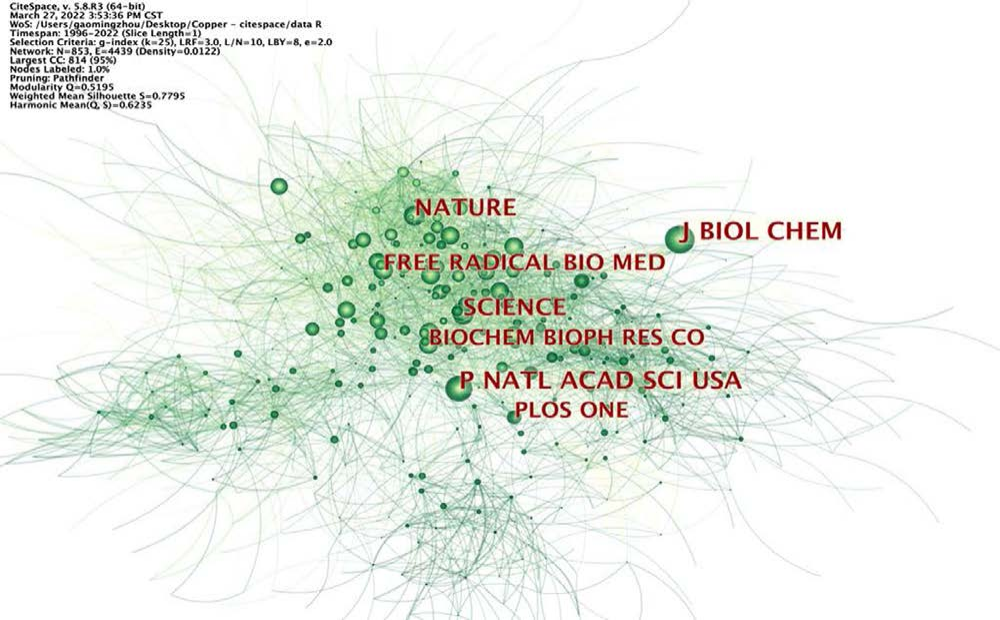
Journal co-citation map on cuprotosis research.

CiteSpace was then applied to generate a co-citation journal map, which resulted in 853 nodes and 4439 links (Fig. 5). Table 3 and Figure 5 shows that the top 10 co-cited journals were *Journal of Biological Chemistry, P Natl Acad Sci USA, Nature, Science, Free Radical Biology and Medicine, Plos One, Biochemical and Biophysical Research Communications, Cell, Cancer Research*, and *Journal of Inorganic Biochemistry*. Moreover, the top 10 journals in terms of centrality were *Journal of Immunological Methods, Cancer Research, Journal of Medicinal Chemistry, Biomaterials, Anticancer Research, Analytical Biochemistry, Nucleic Acids Research, Biological Trace Element Research, Ecotoxicology and Environmental Safety*, and *Chemical Research in Toxicology* (Table 3).

**Table 3:** Top 10 co-cited journals on cuprotosis research with regard to count and centrality.

Rank	Journal	Co-Citation	Journal	Centrality
1	*Journal of Biological Chemistry*	1270	*Journal of Immunological Methods*	0.06
2	*P Natl Acad Sci USA*	1142	*Cancer Research*	0.05
3	*Nature*	893	*Journal of Medicinal Chemistry*	0.05
4	*Science*	891	*Biomaterials*	0.05
5	*Free Radical Biology and Medicine*	752	*Anticancer Research*	0.05
6	*Plos One*	583	*Analytical Biochemistry*	0.04
7	*Biochemical and Biophysical Research Communications*	582	*Nucleic Acids Research*	0.04
8	*Cell*	566	*Biological Trace Element Research*	0.04
9	*Cancer Research*	563	*Ecotoxicology and Environmental Safety*	0.04
10	*Journal of Inorganic Biochemistry*	543	*Chemical Research in Toxicology*	0.04

### 3.4. Analysis of countries and institutions

**Figure 6.**
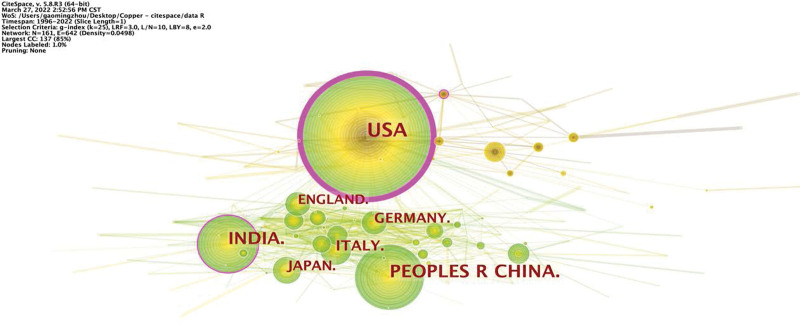
Map of countries involved in cuprotosis research.

Analysis of countries and institutions was conducted to reflect the high-yield countries and institutions in this field. Similarly, CiteSpace generated a country map, resulting in 161 nodes and 642 links (Fig. 6). Results showed that 161 countries produced 2776 publications. The top 10 countries were the USA (709 publications), China (425 publications), India (308 publications), Italy (122 publications), Japan (97 publications), Germany (91 publications), England (86 publications), Brazil (78 publications), Australia (75 publications), and France (74 publications). Furthermore, the top 10 countries in terms of centrality (purple round) were the USA (0.91), England (0.12), India (0.1), China (0.08), Brazil (0.08), FRANCE (0.08), Italy (0.07), Germany (0.05), England (0.05), and Italy (0.05) (Table 4).

**Table 4:** The top 10 prolific countries publishing cuprotosis research in terms of count and centrality.

Rank	Country	Publications	Country	Centrality
1	USA	709	USA	0.91
2	China	425	England	0.12
3	India	308	India	0.1
4	Italy	122	Peoples R China	0.08
5	Japan	97	Brazil	0.08
6	Germany	91	France	0.08
7	England	86	Italy	0.07
8	Brazil	78	Germany	0.05
9	Australia	75	England	0.05
10	France	74	Italy	0.05

**Figure 7.**
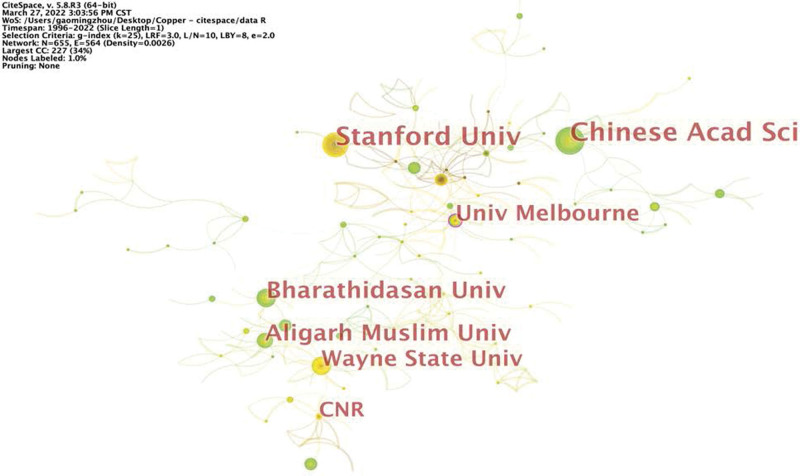
Map of institutions involved in cuprotosis research.

Next, CiteSpace generated an institution map, which resulted in 655 nodes and 564 links (Fig. 7). It was found that the 2776 publications were distributed among 655 research institutions. The top 10 institutions were the Chinese Academy of Science, Stanford University, Bharathidasan University, Aligarh Muslim University, Wayne State University, University of Melbourne, CNR, Indian Institute of Science, King Saud University, and University of Sao Paulo (Table 5).

**Table 5:** Top 10 prolific institutions conducting cuprotosis research.

Rank	Publications	Institution	Centrality	Institution
1	48	Chinese Acad Sci	0.1	Univ Melbourne
2	48	Stanford Univ	0.05	Johns Hopkins Univ
3	31	Bharathidasan Univ	0.05	Karolinska Inst
4	30	Aligarh Muslim Univ	0.04	Wayne State Univ
5	27	Wayne State Univ	0.04	Harvard Univ
6	26	Univ Melbourne	0.04	Natl Inst Technol
7	20	CNR	0.03	Chinese Acad Sci
8	19	Indian Inst Sci	0.03	Bharathidasan Univ
9	40	King Saud Univ	0.03	Indian Inst Sci
10	38	Univ Sao Paulo	0.03	Univ Lisbon

### 3.5. Analysis of authors

**Figure 8.**
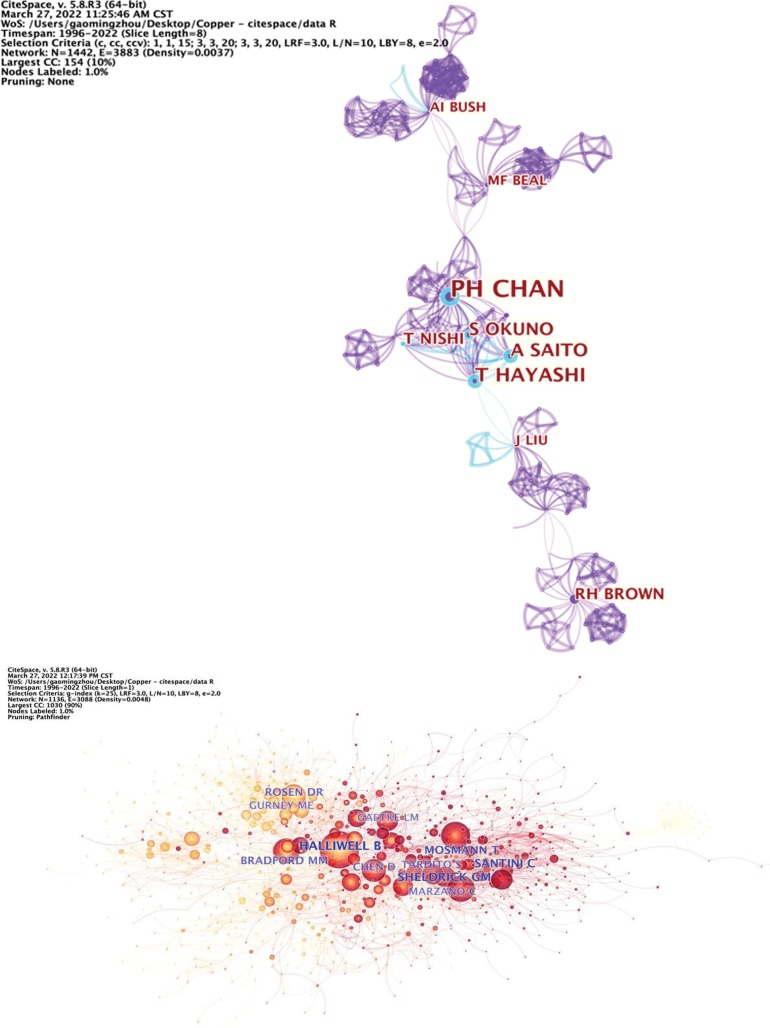
Map of authors that contributed to publications on cuprotosis research.

Analysis of authors could reflect the active authors in cuprotosis research. CiteSpace was applied to generate a coauthor map, which resulted in 1442 nodes and 3885 links (Fig. 8). The top 10 prolific authors who have published cuprotosis research are Ph Chan, Pak H Chan, T Hayashi, Q Ping Dou, Yu Wang, A Saito, Hongjing Zhao, Mingwei Xing, Imrana Naseem, And Nattamai S P Bhuvanesh (Table 6). The top author who owns the highest co-citations is Halliwell B.

**Table 6:** Map of co-cited authors that contributed to publications on cuprotosis research.

Rank	Author	Count	Co-Author	Citation
1	PH Chan	26	Halliwell B	163
2	Pak H Chan	13	Worldhealthorganization	158
3	T Hayashi	13	Sheldrick GM	140
4	Q Ping Dou	12	Santini C	136
5	Yu Wang	12	Mosmann T	119
6	A Saito	11	Bradford MM	98
7	Hongjing Zhao	11	Rosen DR	96
8	Mingwei Xing	11	Tardito S	90
9	Imrana Naseem	10	Chen D	89
10	Nattamai S P Bhuvanesh	10	Marzano C	82

### 3.6. Analysis of references and co-cited references

#### 3.6.1. Analysis of references.

Analysis of references reflects the core reference of cuprotosis research. Table 7 illustrates the top 10 research articles associated with cuprotosis citations. There was a total of 6018 citations among the top 10 most cited research articles. The most cited article, with 1428 citations, was Schutzendubel et al, (2002) titled “Plant responses to abiotic stresses: heavy metal-induced oxidative stress and protection by mycorrhization”. The study explored the mode of action and role of antioxidants as protection from heavy metal stress in roots, mycorrhizal fungi, and mycorrhizae. The theme of “oxidative stress – heavy metals like copper” has attracted significant attention in this research field.^[9]^

**Table 7:** Top 10 co-cited references associated with cuprotosis research with co-citations.

Rank	Citation counts	Reference	First author (yr)
1	1428	Plant responses to abiotic stresses: heavy metal-induced oxidative stress and protection by mycorrhization	Schutzendubel A, 2002
2	715	Metals, oxidative stress and neurodegenerative disorders	Jomova K, 2010
3	570	Functional role of caspase-1 and caspase-3 in an ALS transgenic mouse model	Li MW, 2000
4	536	Transcriptomic footprints disclose specificity of reactive oxygen species signaling in arabidopsis	Gadjev I, 2006
5	513	Copper in diseases and treatments, and copper-based anticancer strategies	Tisato F, 2010
6	497	Induction of nitric oxide-dependent apoptosis in motor neurons by zinc-deficient superoxide dismutase	Estevez AG, 1999
7	478	Slowing of axonal transport is a very early event in the toxicity of ALS-linked SOD1 mutants to motor neurons	Williamson TL, 1999
8	461	Misfolded CuZnSOD and amyotrophic lateral sclerosis	Valentine JS, 2003
9	413	Plasmonic copper sulfide nanocrystals exhibiting near-infrared photothermal and photodynamic therapeutic effects	Wang SH, 2015
10	407	Disulfiram, a clinically used anti-alcoholism drug and copper-binding agent, induces apoptotic cell death in breast cancer cultures and xenografts via inhibition of the proteasome activity	Chen D, 2006

#### 3.6.2. Analysis of co-cited references.

**Figure 9.**
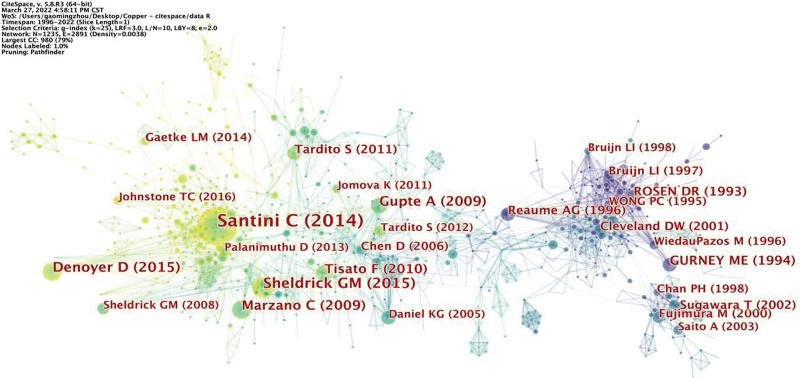
Reference co-citation map associated with cuprotosis research.

Analysis of co-cited references was performed to reflect the research topic. The generation of a co-cited reference map resulted in 1235 nodes and 2891 links (Fig. 9). The most cited reference, with a co-citation count of 133, was “Advances in copper complexes as anticancer agents” published by Santini in 2014. This was followed by Sheldrick 2015, which was co-cited 53 times (Table 8 and 9).

**Table 8:** Top 10 co-cited references associated with cuprotosis research with co-citations.

Rank	Co-citation counts	Co-cited reference	First author (yr)
1	133	Advances in copper complexes as anticancer agents	Santini C, 2014
2	53	Crystal structure refinement with SHELXL	Sheldrick GM, 2015
3	44	Targeting copper in cancer therapy: “copper that cancer”	Denoyer D, 2015
4	41	Elevated copper and oxidative stress in cancer cells as a target for cancer treatment	Gupte A, 2009
5	37	Copper complexes as anticancer agents	Marzano C, 2009
6	35	Copper in diseases and treatments, and copper-based anticancer strategies	Tisato F, 2010
7	31	Motor neuron degeneration in mice that express a human Cu, Zn superoxide dismutase mutation	Gurney ME, 1994
8	28	Mutations in Cu/Zn superoxide dismutase gene are associated with familial amyotrophic lateral sclerosis	Rosen DR, 1993
9	27	Copper-binding agents acting as copper ionophores lead to caspase inhibition and paraptotic cell death in human cancer cells	Tardito S, 2011
10	26	Motor neurons in Cu/Zn superoxide dismutase-deficient mice develop normally but exhibit enhanced cell death after axonal injury	Reaume AG, 1996

**Table 9:** Top 10 co-cited references associated with cuprotosis research with regard to centrality.

Rank	Centrality	Co-cited reference	First author (yr)
1	0.21	Elevated copper and oxidative stress in cancer cells as a target for cancer treatment	Gupte A, 2009
2	0.19	Conversion to the amyotrophic lateral sclerosis phenotype is associated with intermolecular linked insoluble aggregates of SOD1 in mitochondria	Deng HX, 2006
3	0.18	Ceruloplasmin dysfunction and therapeutic potential for Parkinson disease	Ayton S, 2013
4	0.17	Wild-type and mutant SOD1 share an aberrant conformation and a common pathogenic pathway in ALS	Bosco DA, 2010
5	0.16	Affinity gradients drive copper to cellular destinations	Banci L, 2010
6	0.15	Metals and neuroscience	Bush AI, 2000
7	0.15	Prevention of mutant SOD1 motoneuron degeneration by copper chelators in vitro	Azzouz M, 2000
8	0.14	Advances in copper complexes as anticancer agents	Santini C, 2014
9	0.13	Neurodegenerative diseases and oxidative stress	Barnham KJ, 2004
10	0.12	Copper homeostasis and neurodegenerative disorders (Alzheimer, prion, and Parkinson diseases and amyotrophic lateral sclerosis)	Gaggelli E, 2006

**Figure 10.**
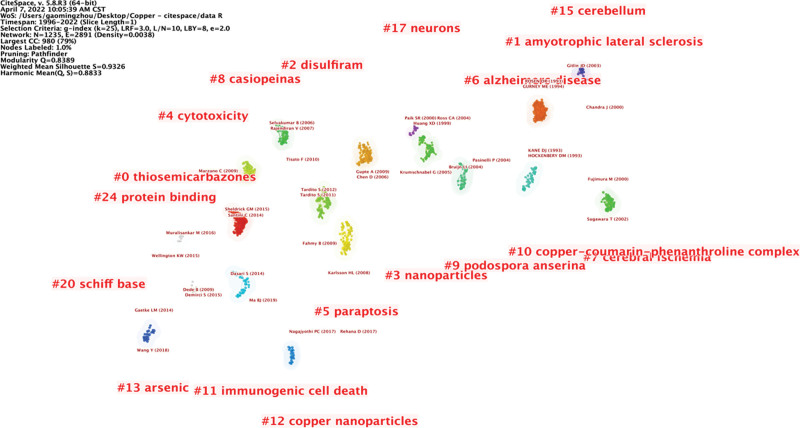
Map of reference co-citation clusters.

The clustering analysis divided the retrieved documents into 19 clusters (Fig. 5). Notably, the literature in each cluster is closely associated with each other and is coordinated in a specific field. The largest cluster was #0, labeled “thiosemicarbazones”, followed by “amyotrophic lateral sclerosis” (cluster #1), “disulfiram” (cluster #2), and “nanoparticles” (cluster #3). Other vital clusters were “cytotoxicity” (cluster #4), “paraptosis” (cluster #5), “Alzheimer disease” (cluster #6), and “cerebral ischemia” (cluster #7), among others (Fig. 10).

### 3.7. Analysis of keyword

#### 3.7.1. Keyword co-occurrence.

**Figure 11.**
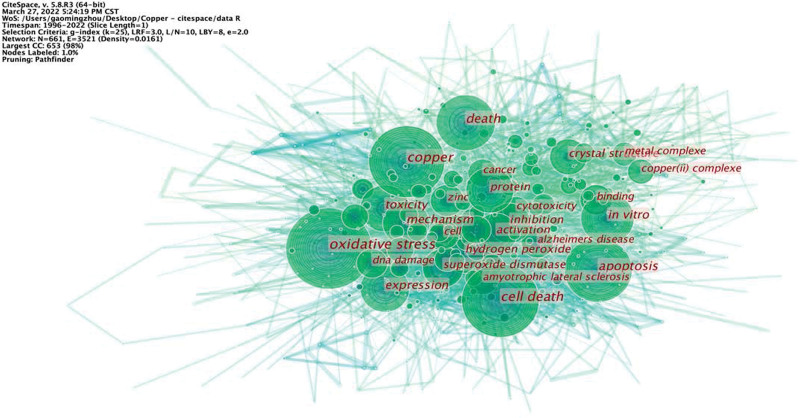
A keyword co-occurrence map of cuprotosis research.

Keyword co-occurrence of a knowledge map reflects the hot topics. Generation a keyword co-occurrence map resulted in 661 nodes and 3521 links (Fig. 11). An analysis of co-occurrence frequency and centrality (Table 10) revealed that the hot keywords were oxidative stress, cell death, copper, apoptosis, death, in vitro, toxicity, mechanism, expression, and crystal structure.

**Table 10:** Top 10 keywords with regard to frequency and centrality in cuprotosis research.

Ranking	Counts	Keyword	Centrality	Keyword
1	531	Oxidative stress	0.09	Disease
2	425	Cell death	0.08	Hydrogen peroxide
3	423	Copper	0.07	Protein
4	322	Apoptosis	0.07	DNA damage
5	250	Death	0.07	Antioxidant
6	231	In vitro	0.06	Activation
7	206	Toxicity	0.06	Cancer
8	195	Mechanism	0.06	Lipid peroxidation
9	195	Expression	0.06	Heavy metal
10	160	Crystal structure	0.06	Brain

DNA = DeoxyriboNucleic Acid.

#### 3.7.2. Burst detection.

**Figure 12.**
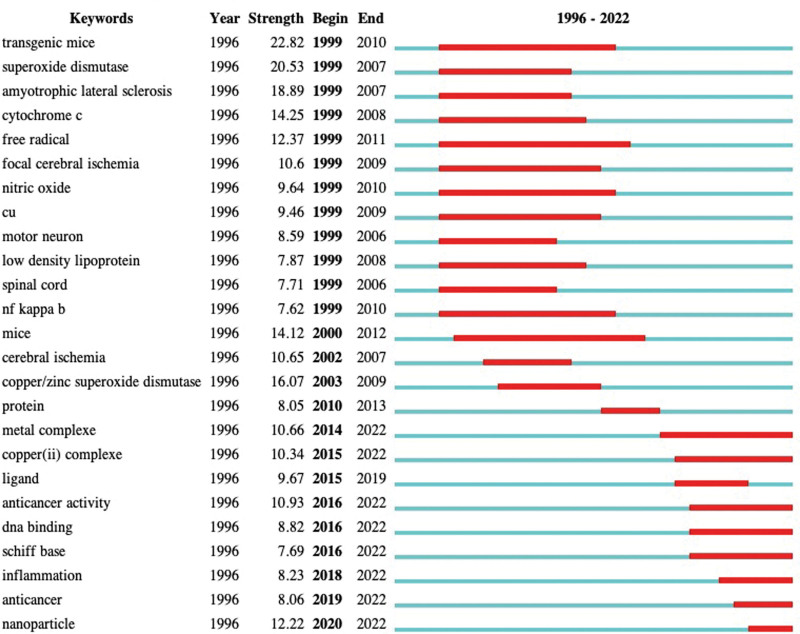
Top 25 keywords with the strongest citation bursts. The red bars indicate keywords cited frequently, whereas the green bars represent those cited infrequently.

Burst detection analysis (keywords that are cited frequently over a period of time) was adapted to identify emergent research frontier concepts. Figure 11 shows the top 25 keywords with the strongest citation bursts in published articles on cuprotosis research. The blue line represents the time interval, whereas the red line refers to the duration of the citation burst. Results showed that transgenic mice were the strongest burst keyword, first appearing in 1999, with a burst strength of 22.82, followed by superoxide dismutase (20.53). It was found that the 8 keywords in the field of cuprotosis research that have impacts on future research are metal complexes, copper complexes, anticancer activity, DeoxyriboNucleic Acid (DNA) binding, Schiff base, inflammation, anticancer, and nanoparticle (Fig. 12).

## 4. Discussion

### 4.1. General status

Copper is a double-edged sword for cells. On the 1 hand, copper is an essential cofactor in all organisms, whereas on the other hand, excessive copper accumulation harms cells. It should be noted that even moderate intracellular copper concentrations can be toxic, ultimately leading to cell death.^[10]^ However, it is not yet known how excess copper induces cell death. Researchers have revealed that the active homeostatic mechanism works through the cross-concentration gradient to keep the intracellular copper concentration at a very low level to prevent the accumulation of free copper in cells. The article “Copper induces cell death by targeting lipoylated TCA cycle proteins”,^[3]^ published in 2022, has attracted significant interest from researchers.

This study found that cuprotosis research could be traced back to 1996. In 1996,

Offiong published “Synthesis and biological activity of platinum group metal complexes of o-Vanillin thiosemicarbazones”,^[11]^ which explored the toxicity of metal complexes. “Copper-induced apoptosis and immediate early gene expression in macrophages” was published in 1999.^[12]^ Since then, research associated with copper-induced apoptosis has exhibited steady growth, with the annual output of research in this field increasing yearly. However, although there has been an upward trend, the study has been marred by occasional fluctuations (Fig. 2). Therefore, cuprotosis will possibly remain a hotspot in the coming years.

Among the main categories in cuprotosis, Biochemistry and Molecular Biology is the most frequently occurring category, indicating that Biochemistry and Molecular Biology are merging organically because of copper research. Conversely, the research category of cuprotosis mainly focuses on the related fields of Biochemistry & Molecular Biology. In addition, we found that the Journal of Inorganic Biochemistry was the most productive journal, with 60 publications on cuprotosis research (Table 2).

Among the 2776 publications identified here, the United States is the country that produces the most articles, with a total of 709 articles (Fig. 6). China is ranked second with 425 publications. An analysis of institutions indicated that the University of the Chinese Academy of Science, located in Beijing, China, has the most achievements in cuprotosis research. Nevertheless, the United States is the core country for cuprotosis research, with the highest centrality of 0.91. The University of Melbourne in Australia is the core institution involved in cuprotosis research, with the highest centrality of 0.1 (Table 4).

Furthermore, it was found that Chan Pak, at Stanford University, is the most prolific author. Notably, his research focuses on neural cell-related death mechanism.^[13,14]^ Halliwell B, who published “Effect of overexpression of wild-type and mutant Cu/Zn-superoxide dismutases on oxidative damage and antioxidant defenses: relevance to Down’s syndrome and familial amyotrophic lateral sclerosis,”^[15]^ had the highest number of citations.

### 4.2. Knowledge base

In bibliometrics, the frontier of a research field represents the current development state of a discipline, and the references in frontier articles constitute the knowledge base of this field.^[16]^ By analyzing the references, we identified the intellectual base in the field of medication literacy. This study examined the references based on the following 2 parameters: citation frequency and centrality. Analysis of references indicated that the current research and discussion mainly focuses on “the relationship between copper and oxidative stress” and “the anticancer mechanism of copper”, including copper complexes as anticancer agents,^[17]^ and elevated copper and oxidative stress in cancer cells.^[18]^ Analysis of reference clusters also indicated that “thiosemicarbazones”, “amyotrophic lateral sclerosis”, “disulfiram”, “nanoparticles”, “cytotoxicity”, and “paraptosis” are the main knowledge base of cuprotosis research.

### 4.3. Research hotspots and trends

#### 4.3.1. Research hotspots.

Generally, high-frequency keywords represent a hot topic in a research field. Herein, keywords analysis revealed 4 main hot topics in cuprotosis research: oxidative stress and antioxidant, the toxicity of copper in vitro, anticancer mechanism, and brain injury in neurological diseases.

##### 4.3.1.1. Oxidative stress and antioxidant

It was found that copper-induced oxidative stress is frequently studied, such as copper-induced oxidative stress in the postlarvae of *Penaeus indicus*,^[19]^ mitochondria-mediated pathway in chicken hepatocytes,^[20]^ and oxidative stress using zebrafish.^[21]^

##### 4.3.1.2. Toxicity of copper in vitro

Given that copper toxicity has always been a research hotspot, the study of copper toxicity in vitro has also gained increasing attention,^[22]^ such as in vitro intestinal toxicity of copper in rat and human cell models.^[23]^

##### 4.3.1.3. Anticancer mechanism

Cancer patients are typically associated with higher copper content in serum and tumor tissues, indicating increased demand for cancer cells for this micronutrient.^[24]^ Consequently, targeting copper has become a research hotspot in the cancer therapy field.^[25]^

##### 4.3.1.4. Brain injury in neurological diseases

The brain is a ravenous user of transition metals, especially copper, zinc, and iron. The destruction of metal homeostasis in the brain is associated with a variety of neurodegenerative diseases, including amyotrophic lateral sclerosis, Alzheimer disease, and Parkinson disease.^[26–28]^

#### 4.3.2. Research trends.

Burst words are considered indicators of research frontier topics over time. Results showed that future research frontiers in cuprotosis research include copper complexes, anticancer activity, DNA binding, inflammation, and nanoparticles. It is worth mentioning that copper is a transition metal, which can exist in oxidation and reduction states. Copper deficiency or toxicity is associated with a variety of pathological conditions. Given the physiological importance of copper and its unique redox activity, many different copper complexes have been synthesized, and their therapeutic and diagnostic potential has been explored in human disease. Currently, the reported copper chelating compounds include clioquinol (5-chloro-7-iodo-8-hydroxyquinoline) (CQ), PBT2, DP-109, tetrathiomolybdate, and pyrrolidine dithiocarbamate.^[29–32]^ A previous study revealed that copper complexes could modulate copper ions homeostasis in the brain, thereby resulting in protective effects in several models of neurodegeneration.^[33]^

Copper is involved as a cofactor in several enzymes, including Reactive Oxygen Species production, tumor progression promotion, metastasis, and angiogenesis. It has also been found at high levels in serum and tissues of several types of human cancers. Therefore, anticancer activity is a research hotspot in the cuprotosis research field.^[34]^ Specifically, synthesizing copper complexes that trigger cell death is one of the strategies for developing novel anticancer copper-based drugs.^[35]^ Biological processes in which copper has been linked to cancer include mitochondrial respiration, collagen cross-linking, immune system modulation, antioxidant defense, mitogenic signaling, and autophagy.^[5]^

However, copper in the bulk form is less available in the body, and much of its amount is excreted out with feces, which causes environmental pollution and economic loss. The application of nanotechnology offers promise to address these issues through synthesizing nanoparticles. Copper nanoparticles not only improve the bioavailability of copper but also have a certain toxic effect.^[36,37]^ Recent studies have shown that copper nanoparticles exhibit antibacterial^[38]^ and anticancer^[39]^ activities. In addition, 1 study reported that mice with lung inflammation were vulnerable to copper-mediated oxidative damage due to copper overload in lung tissue.^[40]^ Copper-binding peptides have also been shown to attenuate microglia inflammation by suppressing the NF-kB pathway field.^[41]^ Consequently, the anti-inflammatory activity of copper is becoming a research hotspot.

## 5. Strengths and limitations

This is the first study to use CiteSpace to perform bibliometric analysis and provide a visual display of publications on cuprotosis research from the cooperation among authors, countries, and institutions to hot spots. However, our study still has some limitations. Since the study was limited to CiteSpace software, we analyzed only English studies in WoS. Therefore, the data may need to be revised. Our results may be inapplicable to research published in other languages. However, this study’s method and data analysis is recognized as sufficient to achieve the research purpose and of great significance.

## 6. Conclusion

This study has shown that cuprotosis research commenced in 1996 and has been conducted to date. Since 1996, the annual output of research in this field has shown an upward trend. Over the past 26 years, the category “Biochemistry and Molecular Biology” has attracted most of the achievements of copper research. Globally, the United States is the core country that produces the most publications, and the University of Melbourne in Australia is the core institution involved in cuprotosis research. Furthermore, Chan Pak at Stanford University is the most prolific author. “thiosemicarbazones”, “amyotrophic lateral sclerosis”, “disulfiram”, “nanoparticles”, “cytotoxicity”, and “paraptosis” form the knowledge base. The main hot topics are oxidative stress and antioxidant, the toxicity of copper in vitro, anticancer mechanisms, and brain injury in neurological diseases. Finally, the future research frontiers are copper complexes, anticancer activity, DNA binding, inflammation, and nanoparticles.

## Author contributions

**Conceptualization:** Liu Li, Xiaoli Xie.

**Formal analysis:** Liu Li.

**Funding acquisition:** Liu Li.

**Investigation:** Liu Li.

**Project administration:** Xiaoli Xie.

**Resources:** Xiaoli Xie.

**Software:** Xiaoli Xie.

**Visualization:** Xiaoli Xie.

**Writing – original draft:** Liu Li.

**Writing – review & editing:** Liu Li.
